# Palp ratio as a field identification tool for two members of the *Anopheles gambiae* complex in Ghana (*A. melas* and *A. gambiae*)

**DOI:** 10.1186/s13071-015-0913-3

**Published:** 2015-05-28

**Authors:** Augustina Angelina Annan, Thomas Florian Kruppa, Ohene Adjei, Rolf Garms

**Affiliations:** Kumasi Centre for Collaborative Research in Tropical Medicine, Kumasi, Ghana; Bernhard Nocht Institute for Tropical Medicine, Hamburg, Germany

**Keywords:** *Anopheles melas*, *Anopheles gambiae*, Palp ratio, PCR.

## Abstract

**Background:**

The *Anopheles gambiae* Giles complex is the most widely studied and the most important insect vector group. We explored the use of the palp ratio method as a field tool to identify *A. melas* and *A. gambiae* in Ghana.

**Methods:**

Human landing catches were conducted to collect mosquitoes in the coastal area of Western Region of Ghana. Palps were removed and segments 3 and 4 + 5 measured using a compound microscope. DNA extraction and downstream PCR for species identification was carried out using the legs and wings. Known *A. gambiae* collected from the Ashanti Region of Ghana were used for comparison.

**Results:**

A total of 2120 *A. gambiae* were collected. Lengths of segments 3 and 4 + 5 were significantly correlated in samples from both regions. Using a palp ratio of 0.81 as the cut-off value, 14.9 % outliers (≥0.81) from our study area were confirmed by PCR as *A. melas.* PCR also confirmed outliers from the Ashanti Region with palp ratio < 0.81 (10.2 %) as *A. gambiae*.

**Conclusion:**

The palp ratio method proved to be a useful tool to identify populations of salt and freshwater *A. melas* and *A. gambiae.*

## Background

The *Anopheles gambiae* Giles complex is the most widely studied and the most important insect vector group. It is presently made up of eight sibling species: *A. gambiae* Giles (formerly molecular S form), *A. coluzzii* Coetzee and Wilkerson (formerly molecular M form), *A. arabiensis* Patton, the salt water breeders *A. melas* Theobald in West Africa and *A. merus* Dönitz in East Africa, *A. quadriannulatus* Theobald in southern Africa, *A. amharicus* Hunt Wilkerson and Coetzee and *A. bwambae* White in Uganda [1]. In Ghana, four species of the complex have been implicated as vectors of malaria; *A. gambiae* in the forest [2,3], *A. melas* at the coast, [4,5] and *A. coluzzii* and *A. arabiensis* in savanna areas [6,7]. *A. melas* and *A. gambiae* are vectors of lymphatic filariasis in coastal Ghana [8,9].

Identification and separation of the sibling species first became possible by using the morphology of polytene chromosomes of salivary gland cells of fourth instar larvae or the ovarian nurse cells of adult females [10]. Later, molecular techniques such as Polymerase Chain Reaction (PCR) were developed [11,12].

As one of the morphological tools, palp ratio (length of the fourth and fifth segment divided by length of the third) has been employed to separate *A. melas* from *A. gambiae* and *A. arabiensis* [13]. Bryan (1980) demonstrated that female *Anopheles* mosquitoes from The Gambia with palp ratios <0.81 and ≥0.81 could be identified as *A. gambiae* and *A. melas* respectively [14]. Using this cut-off point, the number of misidentified specimens was 3.8 % for *A. melas* and 5.8 % for *A. gambiae*. Later, Palsson et al. (1998) compared the palp ratio with PCR results and demonstrated for *Anopheles* from Guinea Bissau that a cut off point of 0.83 correctly identified 100 % of *A. melas*, but erroneously identified 4 % of *A. gambiae* as *A. melas* [15]. Akogbeto and Romano (1999) also reported the presence of *A. gambiae* and *A. melas* from coastal Benin and showed that a cut off ≤0.81 separates *A. gambiae* and >0.81 *A. melas* with an error of 3–6 % [16]. Palsson *et al*. (1998) therefore suggested that the PCR was the most optimal method available to separate *A. gambiae* and *A. melas* and that the palp ratio was not sufficiently reliable [15].

Within the framework of a study on lymphatic filariasis and intensity of transmission in the coastal area of Nzema East (Western Region), Ghana, we investigated whether the palp ratio method can be employed as a field tool to identify *A. melas*. Results of palp measurements were compared with those from *Anopheles* populations from the forest area of the Ashanti Region, Ghana.

## Methods

*A. gambiae* s.l. mosquitoes were collected by Human Landing Catches (HLC) [17] from 18:00 hours to 02:00 hours in 6 villages along the sea coast near Essiama (Nzema East, Western Region) from September 2005 to January 2006. Volunteers (adult males) gave their verbal consent before participating in the collection of mosquitoes. Malaria prophylaxis was given, and treatment (at no cost to the volunteers) was arranged with the local hospitals but none became sick during the study period.

For comparison, *A. gambiae* females were received from two malaria transmission projects conducted in the forest areas of the Ashanti Region in two villages near Agona [2], and sites near Agogo and Konongo [18]. All mosquitoes were stored in cool boxes transferred to a field laboratory in Essiama, and dissected the following day for parity and infections with larval stages of *Wuchereria bancrofti*. The palps were removed, mounted in a drop of 1XPBS on a slide and segments 3 and 4 + 5 measured using a compound microscope. The legs and wings were stored in micro titre plates and transported to the laboratories of Kumasi Centre for Collaborative Research in Tropical Medicine (KCCR). They were stored at -20 °C for later DNA extraction and downstream PCR for species identification [11] and identified as *A. gambiae* s.s. and *A. melas*. The molecular forms M (now *A. coluzzii*) and S (*A. gambiae*) were not separated.

Data were entered using Microsoft Excel. The same programme was used in plotting scattergrams and frequency distributions. STATISTICA for Windows 1993 (StatSoft Inc., Tulsa, OK, USA) was used for the statistical analysis of the results. Correlation coefficients were determined by Pearson Product-Moment correlation and alpha values of less than 0.05 were considered significant.

### Ethical approval

The study was part of a drug trial and treatment study [19] approved by the Ethical Committee of the School of Medical Sciences of the Kwame Nkrumah University of Science and Technology, Kumasi, Ghana. Study procedures were in accordance with the Helsinki Declaration of 1975 (as revised 1983 and 2000).

## Results and discussion

**Fig. 1:**
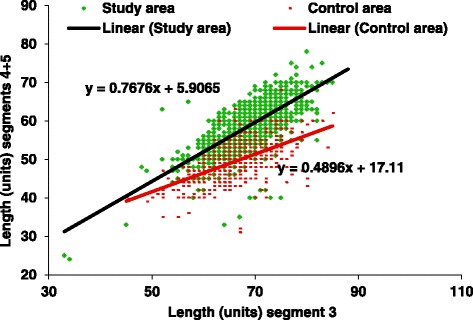
Scattergram showing relations between length of segments 4 + 5 and segment 3 of 1400 *A. melas* from the study area and 856 *A. gambiae* s.s. from the control area

**Fig. 2:**
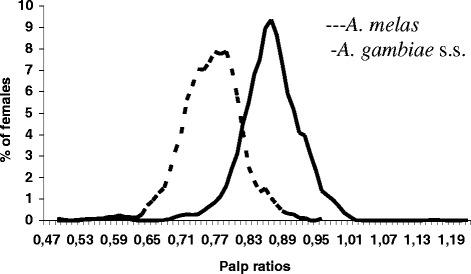
Frequency distributions (average trends) of palp ratios (length segments 4 + 5/length segment 3) of 1264 *A. melas* from the study and 856 *A. gambiae* s.s. from the control area

Lengths of palp segments 3 and 4 + 5 of 1264 *A. gambiae* s.l. from the coastal area and 856 obtained from the forest zone [2,18] were measured. Lengths of segments 3 and 4 + 5 were significantly correlated in both samples (coastal area r = 0.75, p = 0.00, forest zone r = 0.60, p = 0.00, Pearson Product-Moment Correlation). The correlation coefficients of the two groups differed significantly (p = 0.00) indicating that the samples stemmed from different populations (Fig. 1). This was confirmed by the frequency distributions of the ratios of the lengths of palp segments 3 and 4 + 5 (Fig. 2).

The majority of *Anopheles* mosquitoes from the coastal area were classified as *A. melas*. Outliers with palp ratios ≥ 0.81 were confirmed by PCR as *A. melas. A. gambiae* s.s., most likely the S form [5] of the fresh water breeders, is the only species of the *A. gambiae* complex known to occur in the Ashanti Region [2,9,20]. Therefore, using a palp ratio of 0.81 as the cut-off value, 14.9 % *A. melas* from the coastal area as confirmed by PCR would have been wrongly identified as *A. gambiae*. Similarly, using the same cut-off value and PCR, 10.2 % of specimens from the forest area would have been misidentified as *A. melas* (Table 1).

**Table 1:** Comparison of PCR and palp ratio (cut-off 0.81) for identification of putative *A. melas* from the Western Region and *A. gambiae* from Ashanti Region (controls) for species identification

Species	Western Region		Ashanti Region	
	(Study area)		(Control area)	
	PCR (%)	Palp ratio (%)	PCR (%)	Palp ratio (%)
*A. gambiae* s.l.	296	1265	164	856
*A. melas*	296 (100)	1075 (85.1)	0 (0)	87 (10.2)
*A. gambiae* s.s.	0 (0)	189 (14.9)	164 (100)	769 (89.8)

## Conclusion

The palp ratio method proved to be a useful tool to identify populations of salt and freshwater *A. melas* and *A. gambiae* but not sufficiently reliable in identifying individual specimens. In the absence of PCR, especially in resource limited countries where students and scientists do not have access to molecular based techniques, this study recommends the use of the palp ratio to distinguish between *A. gambiae* and *A. melas* as vectors of malaria and lymphatic filariasis in the Ghana coastal area.
